# Recent updates in the surgical treatment of colorectal cancer

**DOI:** 10.1002/ags3.12061

**Published:** 2018-02-15

**Authors:** Takeru Matsuda, Kimihiro Yamashita, Hiroshi Hasegawa, Taro Oshikiri, Masayoshi Hosono, Nobuhide Higashino, Masashi Yamamoto, Yoshiko Matsuda, Shingo Kanaji, Tetsu Nakamura, Satoshi Suzuki, Yasuo Sumi, Yoshihiro Kakeji

**Affiliations:** ^1^ Division of Minimally Invasive Surgery Department of Surgery Kobe University Graduate School of Medicine Kobe Japan; ^2^ Division of Gastrointestinal Surgery Department of Surgery Kobe University Graduate School of Medicine Kobe Japan; ^3^ Division of International Clinical Cancer Research Department of Surgery Kobe University Graduate School of Medicine Kobe Japan

**Keywords:** colorectal cancer, laparoscopic surgery, lateral pelvic lymph node dissection, robotic surgery, transanal total mesorectal excision

## Abstract

Because of recent advances in medical technology and new findings of clinical trials, treatment options for colorectal cancer are evolutionally changing, even in the last few years. Therefore, we need to update the treatment options and strategies so that patients can receive optimal and tailored treatment. The present review aimed to elucidate the recent global trends and update the surgical treatment strategies in colorectal cancer by citing the literature published in the last 2 years, namely 2016 and 2017. Although laparoscopic surgery is still considered the most common approach for the treatment of colorectal cancer, new surgical technologies such as transanal total mesorectal excision, robotic surgery, and laparoscopic lateral pelvic lymph node dissection are emerging. However, with the recent evidence, superiority of the laparoscopic approach to the open approach for rectal cancer seems to be controversial. Surgeons should notice the risk of adverse outcomes associated with unfounded and uncontrolled use of these novel techniques. Many promising results are accumulating in preoperative and postoperative treatment including chemotherapy, chemoradiotherapy, and targeted therapy. Development of new biomarkers seems to be essential for further improvement in the treatment outcomes of colorectal cancer patients.

## 1 INTRODUCTION

Colorectal cancer is the fourth most deadly cancer in the world, because 700 000 patients die of colorectal cancer every year.^1^ Incidence and mortality rates of colorectal cancer are still rising rapidly in most countries except for some of the most developed countries in the world.^2^ To overcome this global disease, new surgical approaches such as transanal endoscopic surgery and robotic surgery are being innovated. Important results of recent clinical trials to elucidate the efficacy of laparoscopic versus open surgery, lateral pelvic lymph node dissection (LLND), preoperative and postoperative therapy, and a watch‐and‐wait approach are accumulating. Several new biomarkers for selecting patients who would benefit by adjuvant therapy are promising. The use of such recent advancements is indispensable for the optimal treatment of colorectal cancer patients.

The present study aimed to elucidate recent global trends and update surgical treatment strategies in colorectal cancer by reviewing the literature published in the last 2 years. Several important studies published more recently are also referred to as essential introductory information.

## 2 SURGICAL TREATMENT

### 2.1 Laparoscopic versus open approach

Although the previous randomized control trials (RCT) showed the superiority of laparoscopic surgery for colon cancer over open surgery in short‐term outcomes,^3^, ^4^ there is critical concern about the feasibility of complete mesocolic excision (CME) or D3 dissection in those trials. Just recently, the outcomes of laparoscopic versus open D3 dissection for stage II or III colon cancer in a randomized control trial (JCOG0404) were reported from Japan.^5^ In this study, the non‐inferiority of laparoscopic surgery to open surgery could not be shown because 5‐year overall survival (OS) of each group was much better than expected (90.4% for open surgery and 91.8% for laparoscopic surgery, *P* = .073 for non‐inferiority). This result suggests laparoscopic D3 surgery could be an acceptable treatment option for patients with stage II or III colon cancer. A meta‐analysis to examine the non‐inferiority of laparoscopic CME or D3 surgery versus open surgery was reported from the United Kingdom (UK).^6^ In their analysis, there was no difference in short‐term mortality and morbidity. Although intraoperative blood loss was significantly less in the laparoscopic group, there was only a trend for longer operative time and shorter hospital stay in laparoscopic surgery compared to open surgery. There was no significant difference in OS and disease‐free survival (DFS). Based on these reports, laparoscopic surgery is considered an acceptable standardized approach for colon cancer even with carrying out CME or D3 dissection.

In contrast, there seems to be some controversy about the non‐inferiority of laparoscopic surgery to open surgery for rectal cancer (Table 1). Two previous large RCT and several meta‐analyses showed similar pathological and oncological outcomes between laparoscopic and open approaches for rectal cancer,^7^, ^8^, ^9^, ^10^ and the laparoscopic approach was regarded as a standardized alternative to the open approach. However, two more recent RCT showed contradictory results, and each failed to show the non‐inferiority of laparoscopic rectal resection to open rectal resection.^11^, ^12^ In the ALaCaRT trial, the number of patients with negative circumferential margin (CRM ≥1 mm) was 222 (93%) of 238 patients in the laparoscopic group and 228 (97%) of 235 patients in the open group (*P* = .06 for non‐inferiority). Primary outcome of successful resection was achieved in 194 patients (82%) in the laparoscopic group and in 208 patients (89%) in the open surgery group (*P* = .38 for non‐inferiority). In the ACOSOG Z6051 RCT, surgical success rate was higher in the open group versus the laparoscopic group (86.9% and 81.7%, respectively, *P* = .41 for non‐inferiority). From Japan, a large cohort study was reported in 2017.^13^ In this study, the proportion of positive CRM cases was not different between the groups (4.53% in the laparoscopic group and 4.47% in the open group), and no significant difference was observed in either 3‐year OS or recurrence‐free survival between the groups. Postoperative complications were significantly less after laparoscopic surgery than open surgery (30.3% vs 39.2%, *P* = .005). The most recent meta‐analysis concluded that the risk of unsuccessful resection in rectal cancer was significantly higher in laparoscopic surgery compared with open surgery.^14^ Therefore, long‐term outcomes are awaited to evaluate whether such poor pathological outcomes have an adverse effect on DFS or OS.

**Table 1:** Laparoscopic vs open surgery for rectal cancer

	COLOR II	COREAN	ALaCaRT	ACOSOG Z6051
Authors	van der Pas et al^8^ Bonjer et al^9^	Jeong et al^7^ Kang et al^10^	Stevenson et al^12^	Fleshman et al^11^
Countries	Europe, Canada, South Korea	South Korea	Australia, New Zealand	USA, Canada
Period	2004–2010	2006–2009	2010–2014	2008–2013
No. patients Lap/Open	1103 739/364	340 170/170	475 238/237	462 240/222
Conversion rate	16.6%	1.2%	8.8%	11.2%
Short‐term outcome	Less blood loss and longer operative time in Lap	Less blood loss and longer operative time in Lap	Less blood loss and longer operative time in Lap	Less blood loss and longer operative time in Lap
Long‐term outcome, 3‐y DFS	Lap: 74.8% Open: 70.8%	Lap: 79.2% Open: 72.5%	NA	NA
CRM involvement	Lap: 56/588 (9.5%) Open: 30/300 (10%) *P* = .850	Lap: 5/170 (2.9%) Open: 7/170 (4.1%) *P* = .770	Lap: 16/238 (6.7%) Open: 7/235 (3.0%) *P* = .06	Lap: 29/240 (12.1%) Open: 17/222 (7.7%)
Incomplete mesorectal excision	Lap: 77/666 (11.6%) Open: 28/331 (8.5%) *P* = .250	Lap: 41/170 (24.1%) Open: 44/170 (25.9%) *P* = .414	Lap: 32/238 (13.4%) Open: 19/235 (8.1%) *P* = .06	Lap: 19/240 (7.9%) Open: 11/222 (5.0%)

CRM, circumferential resection margin; DFS, disease‐free survival; Lap, laparoscopy; NA, not applicable; Open, open surgery.

Another important concern is the indication for laparoscopic surgery in elderly patients with colorectal cancer. A systematic review by Fujii et al^15^ showed significantly better short‐term outcomes of laparoscopic surgery compared with open surgery in terms of estimated blood loss and overall morbidity. They also reported similar long‐term outcomes between laparoscopic and open surgery. Roscio et al^16^ evaluated the effectiveness of laparoscopic surgery for colorectal cancer in the very elderly over 80 years old by a prospective multicenter analysis. They showed similar short‐term and oncological outcomes between laparoscopic and open surgery, and concluded that age was not a risk factor or a limitation for laparoscopic surgery. A large multicenter study in Japan also showed better short‐term outcomes and a lower morbidity rate in the laparoscopic group compared with the open group even in elderly patients with a history of abdominal surgery.^17^ They also showed similar oncological outcomes between the groups. These reports suggest that laparoscopic surgery is safe and is the preferred approach for elderly patients with colorectal cancer.

### 2.2 Robotic surgery

In their systematic review of rectal cancer, Prete et al^18^ reported that robotic surgery had a lower rate of conversion to open surgery, whereas operating time was significantly longer than by laparoscopic surgery. However, perioperative mortality and CRM involvement rate were similar. Another analysis of costs and outcomes between open, laparoscopic, and robotic surgeries of 488 rectal cancer patients showed that operative time was significantly longer in the robotic group.^19^ Estimated blood loss, intraoperative transfusion, length of stay, and postoperative complications were all significantly higher in the open group. Direct cost of hospitalization for primary resection and total direct cost were significantly greater in the robotic group. Huang et al^20^ reported that robotic surgery might offer a shorter learning curve than laparoscopic surgery even in patients who showed more advanced disease after undergoing preoperative chemoradiotherapy (CRT).

In an analysis of abdominoperineal resection, robotic surgery had a significantly lower conversion rate compared with laparoscopic surgery (5.7% vs 13.4%; *P* < .01).^21^ However, it had significantly higher total hospital costs compared with laparoscopic surgery (mean difference, US$24 890; *P* < .01).

Concerning total mesorectal excision (TME) rate, a retrospective analysis of a prospectively maintained database with 20 robotic and 40 laparoscopic surgery cases for rectal cancer was reported.^22^ In this study, the quality of TME was better in the robotic group. In a Japanese retrospective study, 203 robotic surgery cases were compared with 239 laparoscopic cases.^23^ Significantly lower conversion rate (0% vs 3.3%, *P* = .009), less blood loss (15.4 ± 26.4 vs 39.1 ± 85.1 mL, *P* < .001) and shorter hospital stay (7.3 ± 2.3 vs 9.3 ± 6.7 days, *P* < .001) were seen in the robotic group. In contrast, operative time was not significantly different between the groups. Rate of urinary retention was significantly lower in the robotic group than in the laparoscopic group (2.5% vs 7.5%, *P* = .018).

At present, considering the extra financial and time expenses, robotic surgery might be selectively applied for those patients who may benefit from this novel technology.

### 2.3 Transanal TME

Transanal TME (TaTME) was first introduced by Sylla et al in 2010.^24^ Since then, the feasibility and safety of this surgery has been reported by many case studies with acceptable short‐term outcomes.^25^, ^26^, ^27^, ^28^ Most recently, de Lacy's group reported the pathological results of 186 constitutive cases with mid and low rectal cancer.^29^ Complete TME was achieved in 95.7%, and overall positive CRM (≤1 mm) and distal resection margin (DRM) (≤1 mm) were 8.1% and 3.2%, respectively. The international TaTME registry also reported the results of 720 patients.^30^ In 634 patients with rectal cancer, complete TME was obtained in 503 (79.3%), and positive CRM and DRM rates were 2.4% and 0.3%, respectively. Perdawood et al^31^ carried out a retrospective, case‐matched analysis including 300 patients (100 each who underwent TaTME, laparoscopic TME, and open TME, respectively). The CRM positive rate was comparable among the three groups. More favorable outcomes in terms of shorter operation time, less blood loss, and shorter hospital stay were observed in TaTME than in the other two groups. Marks et al^32^ first reported the long‐term outcomes of rectal cancer patients who were treated by TaTME. Rates of successful TME, negative CRM, and negative DRM were 96%, 94%, and 98.6%, respectively. Overall local recurrence, distant recurrence, and 5‐year OS rates were 7.4%, 19.5%, and 90%, respectively. According to a systematic review in 2016, total morbidity of TaTME was 40.3%, which was comparable with that of conventional laparoscopic TME in a previous large RCT.^33^ It showed favorable outcomes of low rates of anastomosis leakage (5.7%) and conversion (3.0%). The rate of positive CRM was 4.7%, and complete TME was achieved in 87.6%. DRM involvement developed in 0.2% only. Importantly, operative and oncological outcomes were better in high‐volume centers (>30 cases in total) than in low‐volume centers (<30 cases in total) including operative time, conversion rate, major complication rate, TME quality, and local recurrence rate. Currently, a multicenter RCT comparing TaTME versus laparoscopic TME for mid and low rectal cancer (COLOR III) is ongoing.^34^

However, technical difficulty of this approach has been well acknowledged by early adopters of this technique. TaTME registry data showed visceral injuries during perineal dissection including five urethral injuries (0.7%), two bladder injuries (0.3%), one vaginal perforation (0.1%), and two rectal tube perforations (0.3%).^30^ The systematic review also detected five cases (0.6%) with urethral injury and five cases (0.6%) with bleeding from the pelvic side wall among 794 patients.^33^

According to a recent survey of the Association of Coloproctology of Great Britain and Ireland (ACPGBI) consultant members, TaTME training was the top educational need for surgeons who wish to start TaTME.^35^ Penna et al^36^ reported the beneficial effect of human cadaveric training courses conducted in the UK and USA. They proposed a structured training curriculum including reading material, dry‐lab purse‐string practice, cadaveric training, and post‐course mentorship as an excellent teaching model for TaTME. Aigner et al^37^ also claimed that the training course on cadavers is indispensable regarding implementation of TaTME into clinical practice. The International TaTME Educational Collaborative Group proposed a detailed framework for a structured TaTME training curriculum including guidance on case selection, multidisciplinary training, mentorship, and assessment.^38^

Although TaTME is one of the most attractive and promising advancements for colorectal surgeons, the risk of adverse outcomes associated with widespread uncontrolled use of this novel technique should be noted. Surgeons are also required to conform to St. Gallen consensus guidelines for safe implementation and practice of TaTME.^39^

### 2.4 Lateral pelvic lymph node dissection

The beneficial effect of lateral pelvic lymph node dissection (LLND) had been under debate for a long time until the results of the JCOG0212 trial were published in 2017.^40^ Five‐year OS, and 5‐year local‐recurrence‐free survival in the mesorectal excision (ME) with LLND and ME‐alone groups were 92.6% and 90.2%, and 87.7% and 82.4%, respectively. Local recurrence rates were 7.4% and 12.6% in the ME with LLND and ME‐alone groups, respectively (*P* = .024). Kanemitsu et al^41^ also reported the outcomes from a total of 1191 consecutive patients with lower rectal cancer who underwent TME with LLND. They described that dissection of the internal iliac nodes and obturator nodes yielded similar therapeutic benefits to those expected from dissection of the superior rectal artery nodes. They also showed that the relative risk for local recurrence was 2.0 for patients with unilateral LLND compared with those with bilateral LLND. Based on these results, ME with LLND is still a standard treatment in Japan. It should be noted that patients with lateral lymph nodes (LLN) larger than 10 mm were excluded and that no patient received any preoperative treatments in JCOG0212.

The effect of additional LLND after preoperative treatment is unclear. Ishihara et al^42^ reported that the incidence of LLN metastasis was estimated to be 8.1% (18/222) even after preoperative CRT. Yamaoka et al also reported that LLN metastasis was detected in seven out of 19 patients who underwent preoperative CRT, suggesting preoperative CRT followed by ME alone is not sufficient, especially when LLN involvement is clinically suspicious.^43^ Ishihara's group carried out TME + LLND for patients with swollen LLN following preoperative CRT.^42^ Akiyoshi's group also carried out LLND with a similar theory and reported 3‐year relapse‐free survival of 75.1% for patients with LLN metastasis.^44^ Currently, RCT to assess the efficacy and safety of LLND after preoperative CRT for rectal cancer patients with suspicious LLN metastases is ongoing in China.^45^

Kusters et al reported that the lateral local recurrence rate was significantly higher in patients with LLN larger than 10 mm than in patients with smaller nodes despite the use of preoperative radiation.^46^ The optimal cut‐off value of LLN size for prediction of metastasis varies among the investigators. Ishibe et al^47^ reported that a cut‐off value of 10 mm was useful for avoiding unnecessary LLND. Akiyoshi's group reported that the optimal cut‐off value before CRT was 8 mm.^44^ Yamaoka reported an optimal cut‐off value of 6.0 mm, with a sensitivity of 78.5% and a specificity of 82.9%.^43^ Before the start of preoperative treatment, accurate estimation of LLN size by MRI is useful.

Although JCOG0212 reported that LLND did not increase male sexual dysfunction, LLND is considered technically challenging.^48^ Recently, the safety and feasibility of laparoscopic versus open LLND was shown by a subgroup analysis of a large multicenter cohort study from Japan.^49^ They also showed similar oncological outcomes between the groups.

Establishment of criteria to accurately predict LLN status as well as standardization of the technique of LLND is necessary in the future.

### 2.5 Preoperative therapy

Currently, fluorouracil‐based chemoradiotherapy is the golden standard of preoperative therapies for locally advanced rectal cancer.^50^ However, the beneficial effect of addition of oxaliplatin to CRT is controversial. In 2016, initial results of the Chinese FOWARC randomized, phase III trial comparing the three arms (fluorouracil‐radiotherapy, n = 155; mFOLFOX6‐radiotherapy, n = 157; mFOLFOX6, n = 163) were reported.^51^ mFOLFOX6‐radiotherapy resulted in a higher pathological complete response (pCR) rate than fluorouracil‐radiotherapy and mFOLFOX6 (27.5% vs 14.0% and 6.6%, respectively). However, higher toxicity and more postoperative complications were observed in patients who received radiotherapy. Long‐term outcomes of the ACCORD12 trial, comparing 45 Gy radiation + capecitabine and 50 Gy radiation + capecitabine + oxaliplatin, reported contradictory results in 2017.^52^ There was no difference between the groups for either DFS or OS. A meta‐analysis including eight clinical trials between 2011 and 2015 was reported in 2017.^53^ Additional oxaliplatin to preoperative CRT significantly improved pCR rate (*P* = .002), decreased local recurrence rate (*P* = .012), and improved DFS (*P* = .000). However, it can increase CRT‐related toxicities, and had no beneficial effects on R0 resection rate, mortality, and OS.

There is increasing interest in a watch‐and‐wait approach as a management option for patients with rectal cancer who received preoperative therapy. The OnCoRe project, which was a propensity‐score matched cohort study, was reported in 2016.^54^ In that project, 129 patients were managed by the watch‐and‐wait approach because of clinical complete response (cCR) after preoperative CRT, and 228 patients who did not have cCR received surgical resection if eligible. Of the 129 patients in the watch‐and‐wait group, 44 (34%) had local regrowth with a 3‐year actual rate of 38%, and three of those 44 patients had synchronous luminal regrowth and distant metastasis. Thirty‐one (76%) of 41 underwent subsequent salvage surgery and five patients (12%) underwent radiotherapy. The 3‐year non‐regrowth DFS rates for the watch‐and‐wait and surgery groups were 88% and 78%, respectively (*P* = .022 by the log‐rank test). The systematic review published in 2017 reported that the regrowth rate in the watch‐and‐wait group was 21.3% and salvage surgery was possible in 93.2% of these patients.^55^ Another meta‐analysis compared the oncological outcomes of the patients who had watch‐and‐wait after cCR versus those who had radical surgery after cCR or versus patients with pCR after surgery.^56^ There was no significant difference among those three groups in terms of non‐regrowth recurrence, cancer‐specific mortality, or OS. However, DFS was better in the patients with pCR identified after surgery compared with the watch‐and‐wait patients (HR 0.47, 95% CI 0.28‐0.78). Although this approach is attractive for patients, confirming long‐term safety in more prospective studies is mandatory.

In contrast, there is a movement to eliminate the use of radiation from preoperative therapy. Results of a study comparing outcomes using the National Cancer Data Base in the USA between neoadjuvant chemoradiotherapy (NACRT) and neoadjuvant multiagent chemotherapy (NAC) for stage II and III rectal cancer were reported.^57^ Although treatment‐related toxicities were not available in that study, the 5‐year OS rate for the NACRT group was significantly better than that of the NAC group (75% vs 67.2%, *P* < .01), suggesting that NAC should not be recommended outside of a clinical trial. FOLFOXIRI is one of the first‐line chemotherapy regimens for metastatic colorectal cancer although it induces high toxicity. So far, only one prospective, phase II study of FOLFOXIRI for resectable colon cancer has been available.^58^ A total of 23 patients with cT4N2M0 colon cancer received FOLFOXIRI followed by surgery. Twenty patients (87.0%) had marked reductions in tumor volume after neoadjuvant treatment. Thirteen patients (56.5%) had grade 3‐4 toxicity, but the toxicity did not affect the subsequent surgery. These results suggest that FOLFOXIRI might be a promising preoperative regimen for resectable colorectal cancer in the future.

For stage IV colorectal cancer with synchronous unresectable metastasis, the impact of primary tumor resection is considered controversial. However, recent large‐scale retrospective studies showed that primary tumor resection with systemic chemotherapy contributed to significantly better overall or cancer‐specific survival compared with chemotherapy alone. In a retrospective cohort study of the National Cancer Data Base from 2004 to 2012, which included 65 543 patients, Maroney et al^59^ reported that primary tumor resection was associated with improved overall survival. Gulack et al^60^ also reported similar results by using the National Cancer Data Base from 2004 to 2012 in a retrospective study of 1446 patients. A recent meta‐analysis by Lee et al^61^ showed patients receiving primary tumor resection plus chemotherapy/radiotherapy had longer overall survival than those treated with chemotherapy/chemoradiotherapy alone (hazard ratio [HR 0.59], 95% confidence interval [CI] 0.51‐0.68; *P* < .001). For a more definitive conclusion, the results of ongoing randomized controlled trials^62^, ^63^ are awaited.

### 2.6 Adjuvant chemotherapy

Although adjuvant chemotherapy for stage II colon cancer patients has been controversial, a recent large‐cohort study including 153 110 patients from National Cancer Data Base in the USA showed that improved OS was associated with adjuvant chemotherapy regardless of treatment regimen, patient age, or high‐risk pathological risk factors in stage II colon cancer.^64^ For rectal cancer, a review published in 2017 concluded that data from the adjuvant rectal cancer trials did not support the use of postoperative adjuvant chemotherapy for patients with rectal cancer treated with preoperative CRT.^65^

Selecting patients who actually benefit from adjuvant therapy is also important. A retrospective analysis using 570 tumor specimens from patients with colon cancer showed that fluoropyrimidine adjuvant chemotherapy benefited patients with stage II or III colon cancer with microsatellite‐stable tumors or tumors showing low‐frequency microsatellite instability (MSI) but not those with tumors showing high‐frequency MSI.^66^ A retrospective pooled analysis using 2141 tumor specimens showed that patients with deficient DNA mismatch repair (MMR) colon cancers have reduced rates of tumor recurrence and improved survival rates compared with proficient MMR colon cancers.^67^ They also showed distant recurrences were reduced by fluorouracil‐based adjuvant treatment in deficient MMR stage III tumors. The MOSAIC study compared fluoropyrimidine monotherapy and fluoropyrimidine and oxaliplatin combination therapy, and hazard ratios for DFS and OS benefit in the combination therapy arm were 0.48 (95% CI 0.20‐1.12) and 0.41 (95% CI, 0.16‐1.07), respectively, in patients with MMR deficiency stage II and III colon cancer.^68^ Unlike fluoropyrimidine monotherapy, fluoropyrimidine and oxaliplatin combination therapy seems to offer a survival benefit for MMR deficiency stage II and III colon cancer patients. Recently, lack of caudaltype homeobox transcription factor 2 (CDX2) expression was identified as a possible prognostic biomarker to predict benefit from adjuvant chemotherapy in stage II colon cancer.^69^ Development of such new biomarkers would allow further progress in adjuvant therapy.

## 3 CONCLUSION

In the present review, we updated advancements mainly in the surgical treatment field of colorectal cancer based on recent important findings. Although surgical technologies including TaTME and robotic surgery are rapidly evolving, surgeons need to carry out their preparations with the most studious care to prevent unfavorable outcomes in patients. Even for laparoscopic surgery, surgeons should keep in mind that recent RCT, the ALaCaRT and ACOSOG Z6051 trials could not show non‐inferiority of laparoscopic surgery to open surgery for rectal cancer in terms of pathological results. The recent progress of preoperative and postoperative treatment is also promising. However, development of new biomarkers seems essential for further improvement in the treatment outcomes of colorectal cancer patients.

## DISCLOSURE

Conflicts of interest: Authors declare no conflicts of interest for this article.
